# Survival time and prognostic factors in canine leishmaniosis in a non-endemic country treated with a two-phase protocol including initial allopurinol monotherapy

**DOI:** 10.1186/s13071-023-05777-2

**Published:** 2023-05-15

**Authors:** Marja Klazina de Jong, Aukje Rappoldt, Femke Broere, Christine Jantine Piek

**Affiliations:** Department of Clinical Sciences, Faculty of Veterinary Medicine, Utrecht University, Yalelaan 108, 3584 CM Utrecht, The Netherlands

**Keywords:** *Leishmania*, Prognosis, Canine, Therapy, Survival, Allopurinol

## Abstract

**Background:**

*Leishmania infantum* is an intracellular protozoan parasite which is endemic in countries of the Mediterranean Basin. Leishmaniosis is increasingly diagnosed in non-endemic areas due to the relocation of dogs from endemic areas and the travel of dogs to and from these areas. The prognosis of leishmaniosis in these dogs may differ from that of those in endemic areas. The aims of this study were (1) to determine the Kaplan–Meier estimated survival time for dogs with leishmaniosis in the Netherlands (a non-endemic country), (2) to determine if clinicopathological variables at the time of diagnosis predicted the survival of these dogs, and (3) to evaluate the effect of a two-phase therapy protocol of allopurinol monotherapy followed by meglumine antimoniate and/or miltefosine in the case of incomplete remission or relapse.

**Methods:**

The database of the Department of Clinical Sciences of Companion Animals of the Faculty of Veterinary Medicine, Utrecht University was investigated for leishmaniosis patients. Patient records were reviewed for signalment and clinicopathological data at the time of diagnosis. Only treatment-naive patients were included. Follow-up was performed during the study by phone contact and included treatment received and date and cause of death. Univariate analysis was performed using the Cox proportional hazards regression model.

**Results:**

The estimated median Kaplan–Meier survival time was 6.4 years. In the univariate analysis, increases in monocyte, plasma urea and creatinine concentrations, and urine protein to creatinine ratio were all significantly associated with decreased survival time. The majority of patients only received allopurinol monotherapy.

**Conclusions:**

Canine leishmaniosis patients in our study population in the Netherlands, which is non-endemic for the disease, had an estimated Kaplan–Meier median survival time of 6.4 years, which is comparable to the outcome of other reported therapy protocols. Increased plasma urea and creatinine concentrations and monocyte concentration were statistically associated with an increased risk of death. We conclude that initial allopurinol monotherapy for 3 months should be effective in more than half of canine leishmaniosis cases, provided there is adequate follow-up, and that meglumine antimoniate or miltefosine therapy should be started as the second phase of the protocol in cases where remission is incomplete or there is a relapse.

**Graphical Abstract:**

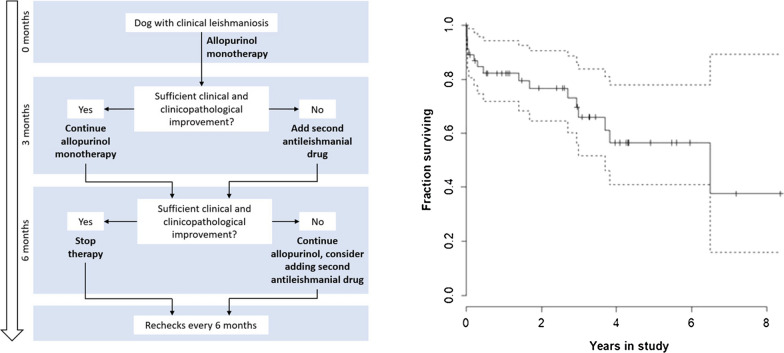

## Background

Canine leishmaniosis is a persistent protozoan infection caused by *Leishmania infantum*. The protozoan is primarily transmitted by its vector, the phlebotomine sandfly. Other modes of transmission, e.g. blood transfusions and bite wounds, are based on incidental reports and scarcely contribute to the overall prevalence [1]. Leishmaniosis is not considered to be endemic in the Netherlands, as its vector is absent from the country [2].

Cases of canine leishmaniosis in the Netherlands mostly occur because of the importation of dogs from endemic countries. The risk that a dog from a non-endemic country develops leishmaniosis after a spending a short time with its owners in southern Europe was calculated to be ≤ 0.23% [3]. The number of dogs imported into the Netherlands in 2018, including those from *Leishmania*-endemic countries, was estimated to be 15,000, which represents approximately 10% of the total number of dogs that are newly acquired in the Netherlands per year. There was a steep increase of > 50% in the importation of (rescue) dogs in 2018 compared to 2016 [4]. As a result, leishmaniosis is progressively increasing in prevalence in the Dutch canine population.

The clinical presentation of canine leishmaniosis varies widely. The clinical signs range from mild dermatitis with only limited systemic disease to severe systemic disease complicated by protein-losing nephropathy. In the Netherlands the most common clinical signs in dogs with leishmaniosis are decreased endurance, weight loss, somnolence, polydipsia and anorexia. The most common abnormalities on physical examination are lymphadenopathy, skin involvement, cachexia and abnormal locomotion [5]. These clinical signs and abnormalities are comparable to those seen in cases in the United Kingdom, which is also non-endemic for canine leishmaniosis [6], and are similar to those seen in endemic countries.

The therapeutic management of canine leishmaniosis consists of prolonged or lifelong monitoring [7], with most dogs requiring intermittent to lifelong therapy. The mainstay therapy for canine leishmaniosis as suggested by expert panels is the combination of allopurinol and meglumine antimoniate [1, 8]. A systematic review of the existing evidence supported the long-term use of allopurinol after an initial course of meglumine antimoniate therapy [9], since it seemed to prevent the early relapse seen when meglumine antimoniate therapy is used alone. The same systematic review concluded that there is insufficient evidence to support the use of allopurinol as a monotherapy [9]. The point estimate for the Kaplan–Meier survival of a cohort of dogs with leishmaniosis in the Netherlands treated with allopurinol was 78% after 6 years (*n* = 21) and 75% after 4 years in a cohort treated with meglumine antimonate [2, 10]. A study undertaken in another non-endemic country, Germany, estimated the survival time of treated and untreated dogs infected with *Leishmania* to be 4.3 years [11]. The efficacy and prognosis of a two-phase treatment protocol as used in the current study, starting with allopurinol monotherapy followed by meglumine antimoniate, and/or miltefosine, in the case of incomplete remission or relapse, has not been examined previously.

The aims of this study were (1) to determine the Kaplan–Meier estimated survival time for treatment-naive dogs with leishmaniosis in the Netherlands, (2) to determine if clinicopathological variables at the time of diagnosis predicted the survival of the dogs, and (3) to evaluate the effect of a two-phase protocol of allopurinol monotherapy followed by meglumine antimoniate, and/or miltefosine, in the case of incomplete remission or relapse.

## Methods

### Patients

In this retrospective cohort study, the patients were initially recruited by identifying all* Leishmania*-positive titers in the database of the University Veterinary Diagnostic Laboratory (UVDL) of dogs presented to the Department of Clinical Sciences of Companion Animals (DCSCA) of the Faculty of Veterinary Medicine of Utrecht University from 2008 to 2017). The patient records were reviewed, and the diagnosis of leishmaniosis was based on abnormalities detected during the clinical examination (e.g. skin lesions including alopecia, desquamation, and ulceration, polyuria/polydipsia, ophthalmic conditions) and/or clinicopathological abnormalities (e.g. anemia, azotemia, elevated total protein) suggestive of leishmaniosis, in combination with a positive anti-*Leishmania* titer and/or cytological confirmation of* Leishmania* amastigotes. Dogs that received treatment with allopurinol, meglumine antimoniate or miltefosine prior to presentation to the DCSCA were excluded from the study. Patient records were reviewed for signalment (including breed, body weight, sex and age), history (including country of origin, travel history) [7], clinicopathological data at time of diagnosis (complete blood count, biochemistry, protein spectrum and urine analysis) and follow-up (including treatment, changes in treatment, and date and cause of death if deceased). For the follow-up period, information was collected from the patient files. Follow-up data were obtained by phone from the referring veterinarian after the dog’s last visit to the DCSCA; the primary goal of this phone call was to gain information on the date and cause of death if the dog had since died.

### Therapy

**Fig. 1:**
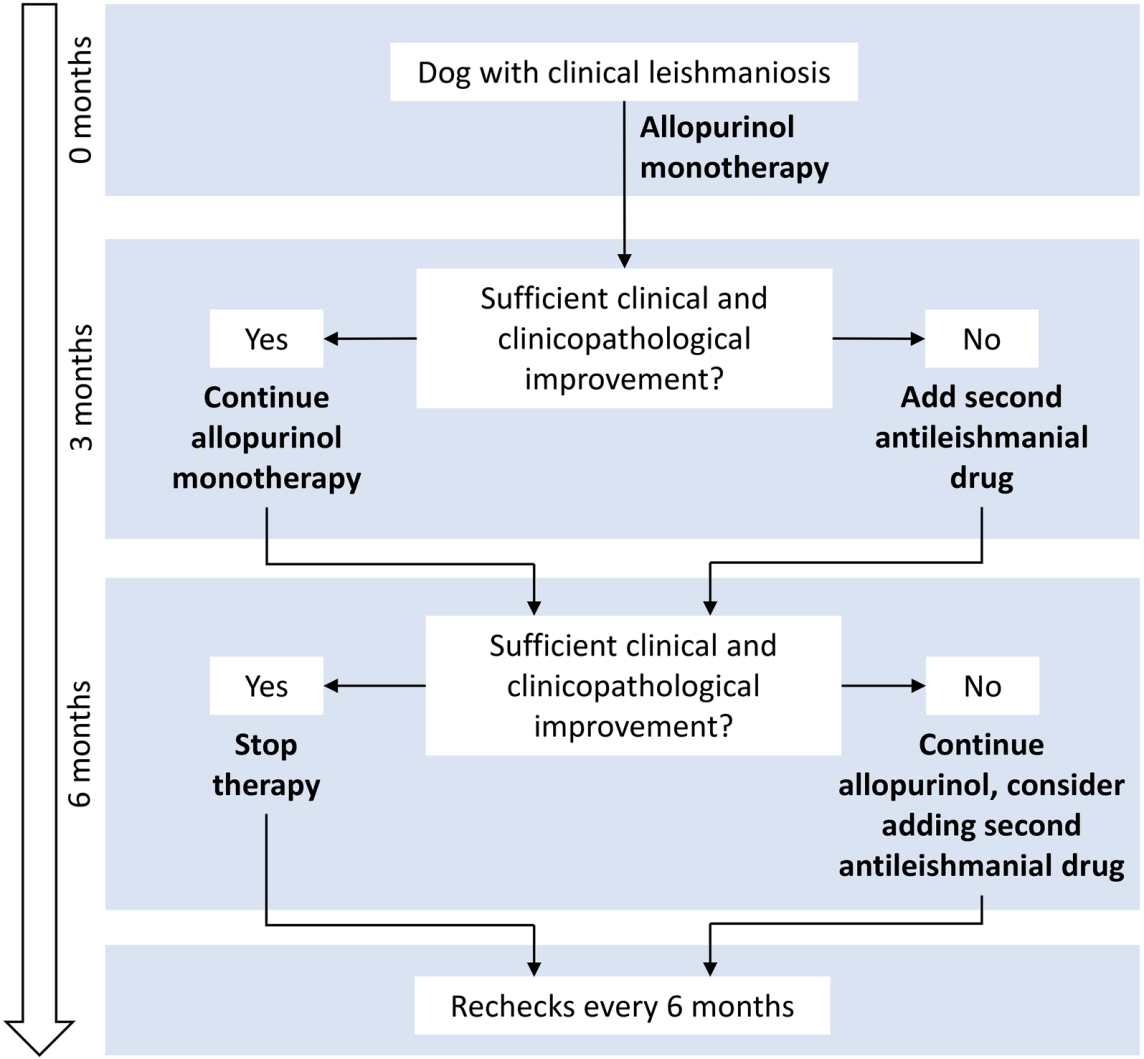
Two-phase treatment protocol. The arrow on the left indicates the timing of scheduled rechecks. If the response based on history, clinical examination and clinicopathological variables was insufficient according to the attending veterinarian at the first recheck (at 3 months), a second antileishmanial drug was added, i.e. meglumine antimoniate or miltefosine. The treatment decision for the second antileishmanial drug was made by attending clinicians based on renal parameters, and also on owner preference. Additional rechecks were planned in severe cases

Treatment choices and changes were at the attending veterinarian’s discretion. The standard two-phase therapy protocol (Fig. 1) included initial treatment with allopurinol monotherapy [minimum 20 mg/kg per day per os (PO)]. If, based on case history, clinical examination and clinicopathological variables, response to initial treatment was insufficient according to the attending veterinarian at the first recheck (at 3 months), additional therapy [meglumine antimoniate 100 mg/kg subcutaneous (SC) every 24 h (q24h) for 3 weeks or miltefosine 2 mg/kg PO q24h for 28 days] was added. Which additional therapy was started depended on the renal function of the dog, as assessed by plasma urea and creatinine levels, urine specific gravity and urine protein to creatinine ratio (UPC), and owner preference. The standard recheck protocol was 3 and then 6 months after starting therapy, after which rechecks were performed every 6 months. However, in the case of severe disease, or in cases with relapse, additional check-ups were planned.

### Laboratory tests

All the tests were performed at the UVDL. All titers for *L. infantum* were measured using a direct agglutination test that had been previously validated for dogs [12].* Leishmania* titer values of < 1:160 are considered negative, 1:320 dubious and > 1:640 (upper limit 1:5120) positive. Complete blood counts were determined on an ADVIA 2120i (Siemens Healthcare, The Hague, the Netherlands), biochemistry on an Olympus AU 680 (Beckman Coulter, Woerden, the Netherlands), urine analysis, including dipstick, sediment and UPC, were determined on an Olympus AU 680 (Beckman Coulter). An UPC > 1.0 was considered to indicate significant proteinuria. The protein spectrum was determined by protein electrophoresis (Hydragel β1-β2, Hydrasis; Sebia, Surrey, UK). Previously determined reference ranges were used.

### Statistical analysis

Descriptive analysis of the clinical pathological data was performed using Microsoft Excel 2010. Reticulocyte percentages were corrected for reference hematocrit by dividing the measured hematocrit by the mid reference value. For thrombocytopenia, the cut-off value was set at 80 × 10^9^/L to diminish the effect of spurious thrombocytopenia. For bicytopenia and pancytopenia, the lower reference range for thrombocytes (144 × 10^9^/L) was used. Creatinine concentrations were corrected for body weight [13]. Survival analysis was performed using the Kaplan–Meier method, including calculating Kaplan–Meier estimated survival times. For each case, survival time was defined as time from diagnosis to event (defined as death by leishmaniosis) or censoring. Animals were censored if still alive at the end of the study, died due to causes unrelated to* Leishmania*, or were lost to follow-up. Univariate Cox regression analysis was used to explore the relationship between clinicopathological variables and Kaplan Meier survival time. For each variable, a choice was made to decide whether a continuous or categorical variable was used. Results were considered significant when *P* < 0.05. Statistical analysis was done using SPSS (IBM SPSS Statistics version 24).

## Results

### Patients

Forty-nine patient records were reviewed. Forty-seven of these dogs fitted the inclusion criteria, whereas two were excluded because of prior treatment for leishmaniosis (Table 1). The median* Leishmania* titer was 1:5120. One dog had a titer of 1:320, three a titer of 1:1280, five a titer of 1:2560 and 38 a titer of ≥ 1:5120. The dog with a titer of 1:320 was positive for* Leishmania* amastigotes in fine-needle aspirates of peripheral lymph nodes. The median age at diagnosis was 3 years (range < 1 year to 11 years). The median weight was 20.8 kg (range 5–47.5 kg). The majority of the dogs were crossbreeds (*n* = 25). Among the 22 pure bred dogs, the most prevalent were Galgo Españols (*n* = 2), Greyhounds (*n* = 2) and Siberian Huskies (*n* = 2); there was one each of 16 other types of pure breed. Thirty-two of the dogs were males (21 neutered) and 15 females (14 neutered).

**Table 1:** Demographics of the study population

Dog identifying number	Age at diagnosis (year)	Sex	Weight at diagnosis (kg)	Breed	Country of origin/visited
1	8	MN	15	Tibetan Terrier	Spain
2	3	FN	27	Crossbreed	Spain
3	3	MN	20	Crossbreed	Spain
4	2	MN	7.1	Crossbreed	Spain
5	5	M	30.4	German Wirehaired Pointer	Spain
6	6	FN	23.09	Galgo Español	Spain
7	3	FN	24.7	Labrador Retriever	Greece
8	1	FN	9.2	Crossbreed	Spain
9	5	FN	15.8	Crossbreed	Spain
10	2	MN	27.1	Crossbreed	Spain
11	3	M	32	Siberian Husky	Spain
12	2	M	47.5	Cane Corso	Spain
13	5	MN	24.1	Greyhound	Spain
14	5	MN	12	Crossbreed	Spain
15	2	FN	6.3	Crossbreed	Unknown
16	2	F	27	German Shepherd	Spain
17	2	MN	28	Galgo Español	Spain
18	11	M	40	Leonberger	Spain
19	8	FN	21.5	Crossbreed	Spain
20	5	M	20.2	Welsh Springer Spaniel	Spain
21	5	FN	25	Greyhound	Spain
22	2	MN	18.4	Crossbreed	Romania
23	2	MN	8.7	Crossbreed	Spain
24	5	MN	9.5	Fox Terrier	Spain
25	3	MN	11.5	Crossbreed	Spain
26	1	MN	16.4	Crossbreed	France
27	8	MN	11.1	Crossbreed	Spain, France
28	8	MN	24.3	Crossbreed	Greece, France
29	8	MN	6.2	Crossbreed	Italy
30	2	FN	29.2	Dobermann	Spain, Aruba
31	6	FN	5	Crossbreed	Spain
32	9	M	28.5	Groenendael (Belgian Shepherd)	Germany, Switzerland, France, Austria
33	5	MN	33.6	Boxer	Spain
34	2	FN	5.3	Poodle	Spain
35	1	M	38	Crossbreed	Turkey
36	6	MN	30.2	Siberian Husky	Greece
37	5	MN	7.9	Shih Tzu	Spain
38	10	MN	17.9	Pointer	Cyprus
39	2	FN	29.9	Mountain dog (unspecified)	Bulgaria
40	5	MN	32.4	Crossbreed	Spain
41	9	FN	29	Crossbreed	Spain
42	1	FN	28.8	Golden Retriever	Unknown endemic country, England
43	1	M	9.2	Crossbreed	Spain
44	0	M	20.8	Crossbreed	Spain
45	3	M	16	Crossbreed	Greece
46	3	M	14.6	Crossbreed	Spain
47	9	MN	7.9	Crossbreed	Spain

*M* Male; *MN* male, neutered; *F* female; *FN* female, neutered

Thirty-nine of the dogs had been imported from countries endemic for canine leishmaniosis. A total of eight of the dogs originated from the Netherlands but had a history of travel to endemic countries. In none of the dogs was the infection considered autochthonous. The most common country of origin was Spain (*n* = 28), followed by Greece (*n* = 4). The countries of origin of the remaining seven dogs included Italy, Romania, Cyprus, Bulgaria and Turkey. The country of origin was not known for two dogs. Of the imported dogs, six had travelled to endemic countries after importation into the Netherlands. The majority of dogs with a history of travel had been to Spain (*n* = 6), of which three had been in Spain regularly (yearly or multiple times a year). Two dogs had a history of travel to France.

Of the 47 dogs, a total of 16 died or were euthanized due to *Leishmania*-related disease, and seven died or were euthanized due to unrelated causes and were censored [cardiac failure (*n* = 2), arthrosis (*n* = 2), suspected intracranial process (*n* = 1), leukemia (*n* = 1), old age (*n* = 1)].

### Therapy

The initial therapy consisted of allopurinol monotherapy in all but one dog. That dog was started on miltefosine monotherapy, followed by the addition of allopurinol 11 days later. Of the 46 dogs started on allopurinol monotherapy, 16 required additional therapy with meglumine antimoniate and/or miltefosine during the period studied. The median time to starting additional therapy was 3.5 months (range 0–23 months). At the end of the period studied, 30 of the 47 dogs had received allopurinol only, 10 allopurinol and meglumine antimoniate, five allopurinol and miltefosine, and two dogs received triple therapy for the duration of their life/follow-up period.

### Laboratory results

A summary of the relevant clinicopathological abnormalities of the dogs at the time of diagnosis is presented in Table 2. About two-thirds (31/46) of the dogs were anemic at the time of diagnosis. Two dogs had severe anemia, 14 moderate and eight mild anemia. In the remainder of the dogs, the hematocrit levels were between the cut-off for mild anemia and the lower reference range [14]. Leukocytosis and leukopenia were present in almost equal proportions, i.e. in six out of 46 and seven out of 46 dogs, respectively. Several dogs (9/45) presented with lymphopenia, and a similar number presented with monocytosis (7/45). Only a few of the dogs (3/46) were considered thrombocytopenic. Similarly, only a few of the dogs were considered pancytopenic (4/46), and about a quarter (12/46) were pancytopenic or bicytopenic. Regarding renal parameters, eight out of 34 of the dogs showed an elevated plasma urea concentration and seven out of 41 of the dogs had an elevated plasma creatinine concentration. Hyperproteinemia was noted in approximately half of the dogs (26/47), with gamma globulins above the reference ranges in 36 out of 44, and alpha 2 above the reference range in two out of 44 of the dogs. Hypoalbuminemia was noted in 24 out of the 47 dogs. Significant proteinuria (UPC > 1.0) was present in 17 out of 35 of the dogs.

**Table 2:** Summary of clinicopathological parameters

Parameter	*n*	Median	Range	Reference range used
Hematology				
Hematocrit	46	0.34	0.10–0.58	0.42–0.61 L/L
Reticulocytes, corrected^a^	26	0.5	0.1–7.5	< 1.5%
Leukocytes	46	7.0	2.0–31.9	4.5–14.6 × 10^9^/L
Segments/neutrophils	45	4.4	0.2–27.4	2.9–11.0 × 10^9^/L
Bands	45	0	0–2.1	0.0–0.3 × 10^9^/L
Lymphocytes	45	1.6	0.1–4.2	0.8–4.7 × 10^9^/L
Monocytes	45	0.4	0–2.7	0.0–0.9 × 10^9^/L
Eosinophils	45	0.2	0–1	0.0–1.6 × 10^9^/L
Basophils	45	0	0–0.2	0.0–0.1 × 10^9^/L
Thrombocytes^b^	46	195	33–572	144–603 × 10^9^/L
Biochemistry				
Urea	34	5.2	2.4–12	3.0–12.5 mmol/L
Creatinine^c^	41	68.8	17.4–470.1	50–129 μmol/L
Total protein	47	75	44–136	55–72 g/L
Albumin	47	25	7–39	26–37 g/L
Alpha 1	44	3	1–8	5–10 g/L
Alpha 2	44	10	5–16	4–13 g/L
Beta 1	44	4	2–9	3–10 g/L
Beta 2	44	10.5	3–54	4–10 g/L
Gamma	44	22.5	5–79	3–9 g/L
Urine analysis				
Urine specific gravity	33	1.027	1.008–1.051	
UPC	35	0.9	0.06–14.14	< 1.0

*n* Number of dogs for which the data were available,* UPC* urine protein to creatinine ratio

^a^Reticulocyte percentages were corrected for hematocrit as follows: reticulocyte × (hematocrit/0.50)

^b^Values < 80 × 10^9^/L were considered to indicate thrombocytopenia

^c^All creatinine values were corrected for body weight; the reference range is a standard reference range

### Statistical analysis

**Fig. 2:**
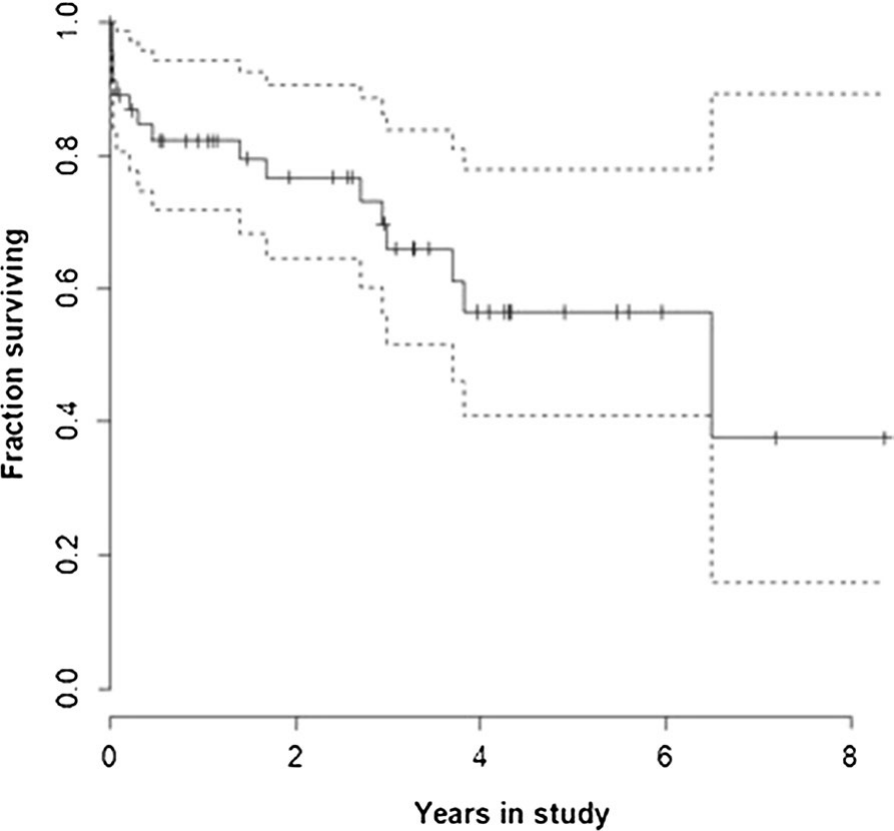
Overall Kaplan–Meier estimated survival curve. The overall Kaplan–Meier estimated median survival time was 6.4 years (95% confidence interval 1.8–11.0 years)

The overall estimated Kaplan–Meier median survival time in the studied population was 6.4 years (77.00 months; 95% confidence interval 21.88–132.11 months) (Fig. 2).

Univariate Cox regression analysis was used to explore the relationships between clinicopathological variables and survival time (Table 3). An increase in monocytes [*P* = 0.018, hazard ratio (HR) = 2.911], urea (*P* = 0.022, HR = 1.057), creatinine (*P* = 0.005, HR = 1.006) and UPC (*P* = 0.010, HR 1.194) was statistically significantly associated with an increased risk of death by *Leishmania* (Table 3).

**Table 3:** Univariate Cox regression analysis

Parameter	*P*-value	HR	Lower 95% CI	Upper 95% CI
Hematology				
Hematocrit	0.088	0.009	0.000	1.998
Reticulocytes (corrected)	0.643	1.268	0.465	3.458
Leukocytes	0.695	1.017	0.936	1.104
Leukocytes (categorical)^a^	0.089	1.739	0.919	3.289
Segmented (categorical)^a^	0.327	1.430	0.699	2.925
Lymphocytes	0.379	0.745	0.386	1.436
Monocytes	0.018*	2.911	1.197	7.079
Eosinophils	0.275	0.272	0.026	2.813
Basophils	0.605	0.063	0.000	2212
Thrombocytes	0.557	1.001	0.997	1.006
Biochemistry				
Urea	0.022*	1.057	1.008	1.111
Creatinine (corrected for BW)	0.005*	1.006	1.002	1.011
Total protein	0.131	0.975	0.944	1.008
Albumin	0.220	0.962	0.905	1.023
Alpha1	0.287	0.842	0.613	1.155
Alpha2	0.276	1.146	0.897	1.466
Beta1	0.655	1.079	0.773	1.507
Beta2	0.208	0.919	0.806	1.048
Gamma	0.425	0.988	0.960	1.017
Urine analysis				
Urine specific gravity	0.446	1.001	0.999	1.003
UPC	0.010*	1.194	1.043	1.367

Parameters were analyzed as continuous variables unless stated otherwise

*HR* Hazard ratio, *CI* confidence interval, *BW* body weight

^a^Categories were below the reference range, within the reference range and above the reference range

* *P* < 0.05

## Discussion

In our cohort of canine leishmaniosis patients in the Netherlands, the estimated Kaplan–Meier median survival time was 6.4 years (95% confidence interval 1.8–11.0 years) (Fig. 2). Survival times are not often reported as outcome parameters in canine leishmaniosis and hence comparison to other studies, including those using different treatment protocols, is difficult. The estimated survival in this study is comparable to the results of two previous studies from the Netherlands. In those studies, the point estimate for the Kaplan–Meier survival of a cohort treated with allopurinol was 78% after 6 years (*n* = 21), and 75% in a cohort treated with meglumine antimonate after 4 years [2, 10]. The median survival time in our study is much longer than that in a Germany study, which estimated the survival time of dogs infected with* Leishmania* to be 4.3 years [11]. This difference might be due to the higher average age and the inclusion of untreated dogs in that study, which showed that treatment in general positively affected survival time in Germany [11]. A positive effect of antileishmanial therapy on survival times has also been found in endemic countries [14]. However, surprisingly little is known about the survival times of dogs with leishmaniosis in endemic countries. Pereira et al. [14] reported survival times of 4.4 and 1.2 years in dogs without and with elevated blood urea levels, respectively [14], but did not report overall survival times in their study, which included both treated and untreated animals. Another study reported survival in treated dogs as ‘long’ when it spanned several years [15]. Guidelines describing prognosis often do not mention survival times but instead use subjective terms such as ‘poor’, ‘guarded’, and ‘favourable’ [1, 8]. Comparison of our data with outcome data reported by others is also difficult due to the use of different outcome parameters, differences in population characteristics, the presence of renal disease in the cohort, differences in treatment protocols, and the possible endemicity of leishmaniosis. However, considering the reported survival times in the literature, our two-phase protocol seems to have given satisfactory results with respect to our study population in the Netherlands.

The second aim of our study was to determine if clinicopathological variables and clinical staging at time of diagnosis predicted the survival of our cohort of treatment-naïve dogs. Increased UPC and plasma urea and creatinine concentrations, and monocytosis were statistically associated with an increased risk of death by leishmaniosis in the cohort in this study. The negative associations of increased plasma creatinine and/or urea, and increased UPC prior to the start of therapy with survival are supported by the results of other studies [10, 11, 14]. In leishmaniosis, antibody-antigen complexes are thought to be the cause of renal damage as they can initiate a chain of events that leads to glomerulonephritis and progressive renal dysfunction [16]. There is also evidence that not only the presence but also the severity of renal compromise affects prognosis, and that in most dogs there is no improvement in renal function during the course of treatment [14]. However, as a prognostic indicator, UPC should be measured prior to the start of therapy since, in some dogs, proteinuria improves with therapy [8, 17]. It is important to note that the presence of renal compromise may have an effect on the choice of therapy, as the use of meglumine antimonate may be contraindicated in cases of renal dysfunction, although there is conflicting evidence for this [8, 18–20]. Monocytosis has not been previously identified as a negative prognostic indicator of leishmaniosis, and our findings contrast with those of other studies in which monocytopenia was associated with a worsening disease status [21, 22].

The third and last aim of our study was to evaluate the effect of the two-phase protocol that comprised initial allopurinol monotherapy with the addition of meglumine antimonate or miltefosine when relapse occurred or clinical remission was not achieved within 3 months. In 67% of the dogs (31/46) in this cohort, allopurinol monotherapy led to complete clinical remission and the dogs did not need any additional antileishmanial therapy (meglumine antimonate or miltefosine) for the rest of their lives. In those dogs that were non-responders or had a relapse, complete remission was achieved by treatment with meglumine antimonate and/or miltefosine. A randomized clinical controlled trial that evaluated allopurinol monotherapy showed that it led to remission of clinical signs and normalization of clinicopathological abnormalities [23]. Similarly, two studies in which allopurinol was examined as a monotherapy reported complete clinical remission in 50–100% of dogs within a period of 3–6 months in cohorts of 21 [2] and 19 dogs [24]. Our results support the findings of other studies that a longer remission period is possible [25]. We conclude that, provided that the effects of the therapy are monitored appropriately, the two-phase protocol described in this study is a good means of adjusting the therapy to the needs of individual dogs, with good clinical results. In line with this, the results of questionnaires indicate that many veterinarians use allopurinol monotherapy as a mainstay in the management of leishmaniosis in endemic countries [26, 27], as is also commonly the case in non-endemic countries [2, 11]. This differs from published guidelines [1, 28] and the conclusions of the most recent systemic review on the treatment of canine leishmaniosis [9]. The results of our study, however, indicated that over 50% of the dogs in our study population of a non-endemic country survived for at least 6 years when treated with allopurinol monotherapy to which meglumine antimonate and/or miltefosine was added in the two-phase protocol if considered necessary.

An observational study without a comparison group, like the current study, can be classified as a retrospective cohort study and may provide the highest level of evidence for assessing prognosis in the study population. Additionally, prospective studies where the focus is on determining long-term survival are difficult to undertake. Because of the retrospective nature of this study, the data analysis was focused on robust parameters such as laboratory test results, date of death and change of therapy, rather than on clinical recovery, as major outcome data. Selection bias was minimized by selecting cases based on strict diagnostic criteria, and only treatment-naive cases were included. All of the dogs in the study had received antileishmanial drugs after the diagnosis of the disease. Because of the retrospective nature of this study, the possibility of co-infections with other vector-borne disease that had a negative impact on survival time could not be ruled out in all cases. The onset of clinical signs, as recorded at the time of diagnosis, was often unclear from the files, and this may have affected the outcome parameters. Although attrition bias was managed by censoring cases that died from causes other than leishmaniosis, it may have occurred inadvertently as pathologic investigations were not undertaken for the 23 dogs that died during the study. Dogs were censored after careful evaluation of the information on them in the files. However, in the absence of a post mortem examination, it is difficult to establish if death, or the decision to euthanize a dog, was influenced by the *Leishmania* infection. Chance, another major source of error, is unlikely to explain the results, as the outcomes of this study are comparable to those of other studies, as discussed above.

## Conclusions

The canine leishmaniosis patients in our study population in the Netherlands, which is non-endemic for the disease, had an estimated Kaplan–Meier median survival time of 6.4 years. Increased plasma urea and creatinine concentrations, an increased UPC and higher monocyte levels were statistically associated with an increased risk of death. We conclude that more than half of the canine leishmaniosis cases in our study population were adequately managed by the allopurinol monotherapy that was given at the beginning of the two-phase protocol. For the remainder of the dogs, the protocol included the addition of meglumine antimoniate or miltefosine therapy.

## Data Availability

The data supporting the conclusions of this article are included in the article. Raw data are available for researchers upon request.
